# Context-dependent alarm responses in wild vervet monkeys

**DOI:** 10.1007/s10071-023-01767-0

**Published:** 2023-03-17

**Authors:** Adwait Deshpande, Erica van de Waal, Klaus Zuberbühler

**Affiliations:** 1 Department of Comparative Cognition, Institute of Biology, University of Neuchatel, Neuchatel, Switzerland; 2 Inkawu Vervet Project, Mawana Game Reserve, KwaZulu-Natal, South Africa; 3 Department of Collective Behavior, Max Planck Institute of Animal Behavior, Constance, Germany; 4 Centre for the Advanced Study of Collective Behaviour, University of Konstanz, Constance, Germany; 5 Department of Ecology and Evolution, University of Lausanne, Lausanne, Switzerland; 6 School of Psychology and Neuroscience, University of St Andrews, St Andrews, UK

**Keywords:** Evolution of language, Vocal communication, Nonhuman primates

## Abstract

The alarm calls of nonhuman primates are occasionally cited as functionally equivalent to lexical word meaning in human language. Recently, however, it has become increasingly unlikely that one-to-one relations between alarm call structures and predator categories are the default, mainly because many call types are produced in multiple contexts, requiring more complex notions of meaning. For example, male vervet monkeys produce the same alarm calls during encounters with terrestrial predators and neighbouring groups, suggesting that recipients require additional information to attribute meaning to the calls. We empirically tested the hypothesis that vervet monkeys take contextual information into account when responding to each other's alarm calls. In playback experiments, we exposed subjects to recordings of male alarm barks during actual intergroup encounters (predator unlikely) or when there was no intergroup encounter (predator likely). Subjects responded more strongly in the no intergroup encounter situations, typically associated with discovering a hiding predator, measured in terms of startle responses, vigilance behaviour and gazing towards the presumed caller. We discuss the significance of using contextual information for meaning attribution in nonhuman primate communication.

**Supplementary Information:**

The online version contains supplementary material available at 10.1007/s10071-023-01767-0.

## Introduction

A significant milestone in the Darwinian quest for evolutionary continuities in cognition (Darwin 1872) has been the discovery that vervet monkeys (*Chlorocebus pygerythrus hilgerti*) produce acoustically distinct alarm calls to refer to different types of predators (Struhsaker 1967), which has led to the suggestion that animal alarm calls can function as if they possessed lexical meaning (Macedonia and Evans 1993; Seyfarth et al. 1980a). In animal communication, the evidence is typically in the form of acoustically distinct calls produced in context-specific ways, in analogy with lexical semantics in linguistics with mental representations referring to the meaning of words. For example, monkeys give 'eagle' alarm calls to particular species of raptors, or 'snake' alarm calls given to some dangerous snake species (Pereira and Macedonia 1991; Zuberbühler 2000a, 2001; Manser 2001; Kirchhof and Hammerschmidt 2006; Cunningham and Magrath 2017).

However, with the advancement of acoustic analysis techniques and long-term behavioural observations, the tight one-to-one links between acoustically distinct alarm calls and their eliciting predator contexts have mostly dissolved. What initially looked like a predator-specific alarm call turned out to be a call given to multiple situations, often with no obvious underlying coherence (Price et al. 2015). But why would natural selection favour the evolution of contextually ambiguous alarm signals in the predation context? If the alarm calls do not refer to specific types of danger, how can recipients ever make adaptive anti-predator decisions, one of the presumed functions of animal alarm calling?

It is important to remember that the predator fauna often varies within a species' geographic range and can also change rapidly over time, which requires considerable mental flexibility by the recipients. One solution to the conundrum is that recipients attend to contextual information beyond the acoustic structure of the calls themselves to make inferences about the most likely cause of an alarm call (Fischer and Price 2017). A few empirical studies have already provided relevant evidence for the influence of context on call inference. First, putty-nosed monkeys show less vigilance to terrestrial alarm calls if the calls are preceded by the sound of a falling tree compared to no preceding cues (Arnold and Zuberbühler 2013). In addition, Diana monkeys respond differently to the playback of Guinea fowl alarm calls, depending on the priming of different predator types before the playback (Zuberbühler 2000a, b, c).

Of course, it is always possible that what appears to be one call type is, in fact, a group of subtle, acoustically distinct call variants, which refer to separate external events and so facilitate meaning attribution for recipients. In line with this, call variants appear to be a fairly frequent phenomenon in primate communication, with evidence in Campbell's monkey alarm calls (Keenan et al. 2013) or chimpanzee soft ‘hoos’ (Crockford et al. 2018). Like humans, some primates appear to be able to perceive acoustically graded signals in categorical ways (e.g., Barbary macaques: Hammerschmidt and Fischer 1998), adding plausibility to the acoustic variant hypothesis.

Here, we revisit the alarm call system of vervet monkeys, the classic example of ‘lexical semantics’ in animal communication. In the South African subspecies (*Chlorocebus pygerythrus pygerythrus*), males have been observed to produce alarm barks to terrestrial predators but also during within- and between-group aggression (Price et al. 2015). Thus, the male alarm bark in this subspecies is an ideal call type to investigate whether listeners take additional information into account to make inferences about a call's meaning. We experimentally tested how free-ranging South African vervet monkeys responded to male alarm barks when primed with or without intergroup encounters. We used naturally occurring intergroup encounters and no ongoing intergroup encounter situations as contexts, during which we played back recordings of male alarm barks in controlled ways.

Encounters between two neighbouring monkey groups are regular events ranging from peaceful co-foraging to severe physical aggression (Willems et al. 2015). Interestingly, during aggressive encounters, males from both groups sometimes produce alarm barks that acoustically resemble terrestrial predator alarms, along with other aggressive vocalisations (Price et al. 2015). The responses to these alarm barks during aggressive encounters are varied and sometimes include calls by other males. Participation in intergroup aggression varies from case to case but is most common for high-ranked individuals, although it also depends on prior social interactions (Willems et al. 2015; Arseneau et al. 2016, 2018). As a result, different subgroups of individuals participate in frontline aggression during each encounter, while others stay back and appear uninterested.

We took advantage of this fact to examine whether disengaged individuals used contextual information when hearing the male alarm barks of frontline males during between-group encounters. To control for the acoustic variant hypothesis, we recorded male alarm bark variants originally produced during terrestrial predator encounters or between-group encounters as playback stimuli. We predicted that if non-participating and uninterested individuals took context into account, they should show differential (strong or weak) responses to the playback of alarm barks presented during ongoing between-group encounters compared to when there was no ongoing encounter. Alternatively, if the context played no role in how an individual responded to the alarm bark, we expected no significant difference in responses across treatments. Finally, if the call variant alone drove the responses, we predicted that the subject would respond differentially on the basis of the call variants they heard, regardless of the context. We discuss our results in light of different explanations for the use of contextual information.

## Methods

### Study site and groups

The study was conducted between July and November 2019 at the Inkawu Vervet Project (IVP), located in the Mawana Game Reserve, Kwazulu Natal, South Africa (S 28° 00.327; E 031° 12.348). All observers complete a standard training protocol and complete inter-observer reliability and individual identification tests before participating in data collection. Researchers have access to several neighbouring groups of wild South African vervet monkeys that are well-habituated to human presence and allow close observations by multiple observers. Data for this study were collected from one group (BD), consisting of 57 individuals (adult females: *N* = 16; adult males: *N* = 12; juvenile females: *N* = 15; juvenile males: *N* = 14).

### Experimental procedure

We tested the effects of alarm bark variants on subjects engaged in either a between-group encounter (BGE trials, *N* = 8) or no between-group encounter (non-BGE trials, *N* = 8), using a within-subject design (*N* = 8 adult female subjects; *N* = 16 trials).

**Fig. 1:**
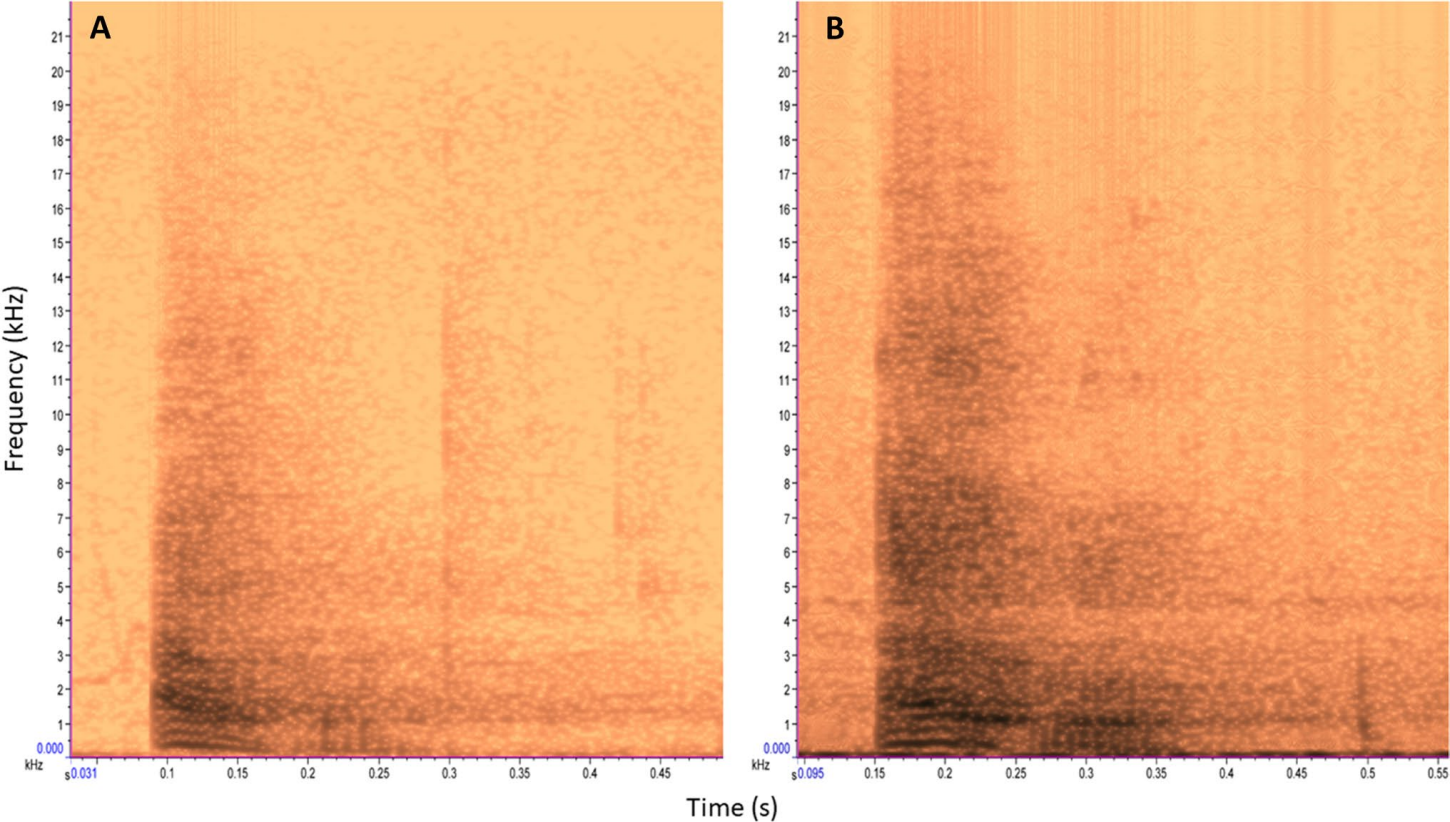
Spectrograms (Hamming window at 1024 DFT and 93.8% overlap) of the exemplars of calls from a BD male used as playback calls for the experiment **A** PRE variant: Alarm bark recorded during a terrestrial predator encounter **B** BGE variant: Alarm bark recorded during between-group encounters

We recorded alarm barks produced by adult males of three groups in both between-group encounter (BGE variant) and terrestrial predator encounter (PRE variant) situations. Calls were acoustically highly similar (see Supplementary material for acoustic) (Price et al. 2015; Besson 2017). Out of all the call recordings, we selected one BGE and one PRE variant produced by a single adult male in the BD group who was present in the group for the entire duration of the experiment as playback stimuli for the experiment (Fig. 1). Both exemplars were from the same adult male and were the only ones in our database with the sufficiently high acoustic quality required for playbacks. We randomly assigned the BGE variant as a playback stimulus to four subjects and the PRE variant to the remaining four subjects. We used the same call variant for BGE and non-BGE trials for each subject.

**Fig. 2:**
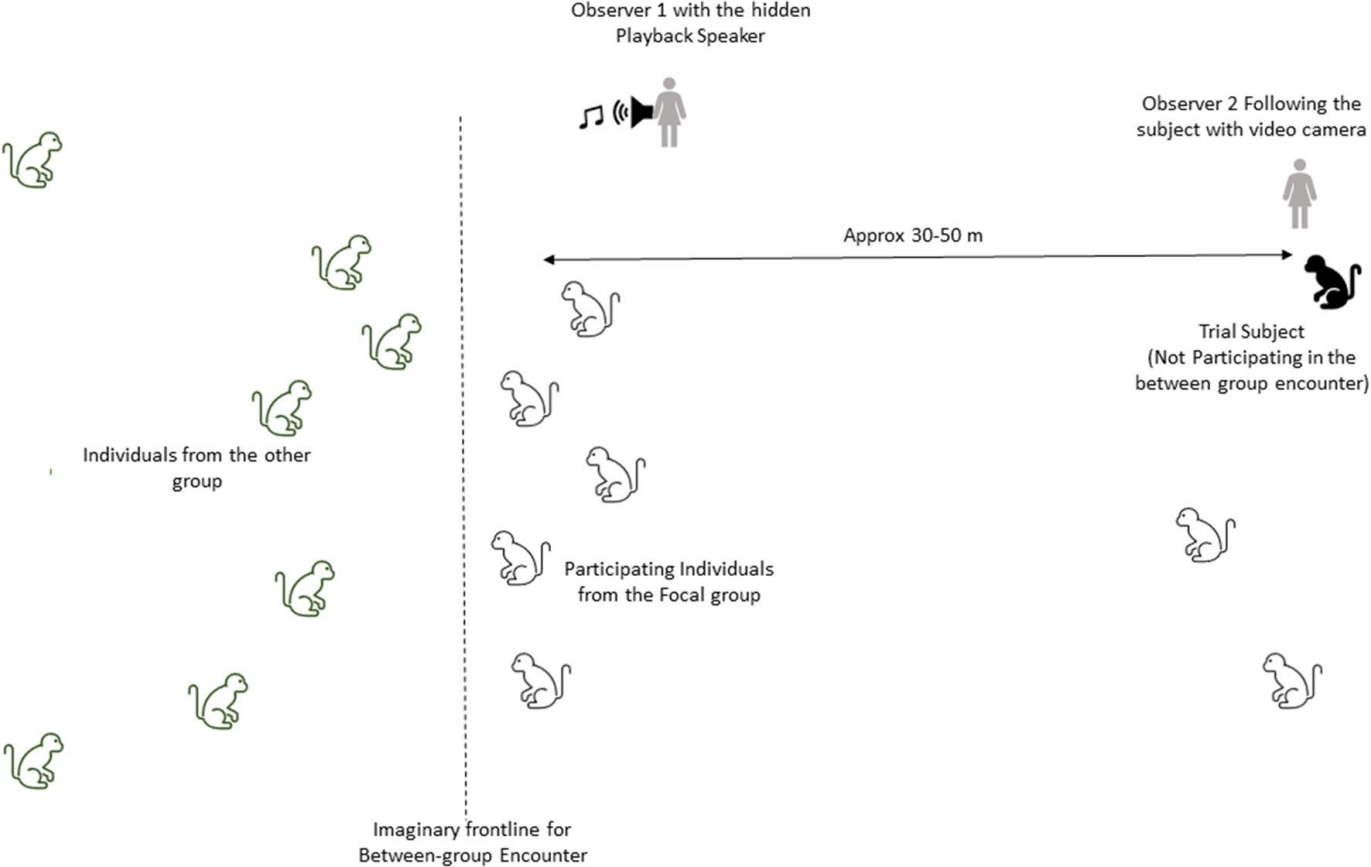
Schematic illustration (not to scale) showing BGE trial design

We removed background noise and padded the calls with two seconds of silence before and after the utterances using Raven Pro 1.5 (Center for Conservation Bioacoustics 2014). Recordings were played back using a Motorola XPlay phone and an Anchor AN-MINI-RST-01 speaker. Call amplitudes were adjusted and kept constant across trials, such that they matched natural calls at a distance of 30–50 m which was also the usual distance between subjects and BGE frontline during BGE trials (see below) (Fig. 2).

### BGE trials

Since between-group encounters occurred unpredictably, we conducted BGE trials opportunistically, provided the following conditions were met. First, we only initiated a trial after visible intergroup aggression. This was because male alarm barks were usually given during intergroup encounters that involved physical aggression between the two groups. The caller usually actively participated during intergroup aggressions and most likely remained on the frontline (see below). Second, previous studies (Ducheminsky et al. 2014) suggested that habitat type and the group spread affected responses to alarm calls, so we only conducted experiments in mixed habitat type (i.e., canopy cover > 50% to < 100%) and the group spread approximately 100 m (i.e., the distance between the subject and all other group members no more than 100 m).

An intergroup encounter started when members of another group were within 50 m of any individual of the study group (BD) or if BD individuals were vocally reacting to another group with grunts, screams or chutter calls (Cheney and Seyfarth 1982). All eight trials were conducted when BD encountered either habituated group of AK (*N* = 5) or a semi-habituated group CR (*N* = 3), which made it possible to observe monkeys without interfering with their behaviour. Once the encounter had started, we waited for at least 5 min before carrying out a trial. We only conducted a trial if there were between-group aggressive interactions (running towards the opposing group, aggressive vocalisations, chasing, grabbing, and biting individuals of the opposing group) during this period. However, if any male from either group produced an alarm bark during this period, we aborted the trial. We also made sure the caller male of the playback call was not present in the vicinity (approx. 10–15 m) of the subject of the trial and not directly visible to the subject.

Two observers were present during each experiment. The first observer positioned herself on the front line of the encounter, i.e., the imaginary line that separated the two groups, where most between-group interactions happen (Arseneau et al. 2016, 2018; Willems et al. 2015). All individuals within 10 m of the front line were considered participants in the encounter. Frontlines could shift during encounters, particularly when one group decides to retreat (Fig. 2). The first observer continuously monitored the frontline and also operated the playback speaker.

The second observer selected and monitored the subject provided it was not participating in the encounter (i.e., located at least 30 m from the front line, not showing any interest in the encounter but engaged in foraging, resting or grooming). The subject was continuously filmed using a JVC quad-proof EverioR camcorder. Both observers communicated with each other via Motorola radios, and once all conditions were met, the first observer played a male alarm bark variant from the speaker near the front line. The second observer video-recorded the subject's behaviours for the next 10 min from the onset of the playback. The average time gap between the two consecutive BGE trials on different subjects was 8.7 days.

### Non-BGE trials

The same individuals were tested during non-BGE trials with the same playback stimulus with a minimum gap of three days from the BGE trials, but this time in the absence of intergroup encounter, when most group members were either foraging, resting or grooming. We ensured that no intergroup encounter and no natural alarm-calling event occurred for at least 2 h before a trial. As with BGE trials, we only conducted non-BGE trials in mixed habitats and with a group spread of about 100 m. The second observer located the subject and followed it for at least 10 min. If no relevant event occurred during this time period (participation in intra-group aggression), the second observer informed the first observer to start the trial. Similar to BGE trials, the first observer then positioned herself between 30 and 50 m from the subject hidden behind a bush to broadcast the playback stimulus upon the instructions from the second observer on the radio. The second observer video-recorded the subject continuously for 10 min from the onset of the playback call.

### Behavioural responses

For both types of trials, the second observer continuously scored the overall group activity, the number of individuals present within 5 m of the subject before each trial (audience size), and any other significant behaviours by the group members.

We coded key behaviours during the first minute following each playback trial using BORIS V. 7.10.2 coding software (Friard and Gamba 2016). We were specifically interested in the following behaviours by the subject: (1) Looking towards the speaker (duration of looking in the direction of the speaker (s), as inferred from head orientation); (2) vigilance [duration of scanning environment with alert body posture (s)]; (3) startle [sudden complete or upper body jerk movement, with a rapid head movement in any direction (yes/no)] (Ducheminsky et al. 2014); (4) predator-specific responses (running into the nearest tree or bush for terrestrial predator (yes/no) (Seyfarth et al. 1980b, c).

To assess inter-observer reliability, 8 out of 16 trial videos were randomly selected and scored by a second coder blind to the hypotheses and objectives of the study but familiar with primate behaviour. We then compared the key behaviours described above between the two observers. As the direction of the speaker was not obvious in the video to the naïve coder, he was informed about the direction of the speaker at the start of the video. We calculated Pearson's correlations coefficients and ran paired *t* tests between two coders for the continuous variables (Looking towards the speaker: *r* = 0.78, *p* = 0.02, *t* = 0.05, *p* = 0.96; Vigilance: *r* = 0.88, *p* = 0.003, *t* = 0.90, *p* = 0.39) and Cohen's kappa was calculated for the startle responses (*k* = 0.91). Both coders agreed that no predator-specific responses occurred. Thus, the inter-observer agreement was sufficiently high for all three key behaviours.

### Statistical analyses

All statistical analyses were conducted in R v4.0.3 (Team 2020). We used non-parametric repeated measures tests to verify the effects of treatments and call variants used as playback stimuli on the response variables (Duration of looking towards the speaker, vigilance, and startle response). To test our prediction that both duration of looking towards the speaker and vigilance is lower in the BGE trial, we used one-tailed paired Wilcoxon signed rank tests. Similarly, to test the effect of call variants, we used two-tailed paired Wilcoxon signed rank tests. We also calculated effect sizes for all the tests.

We could not use the same approach to assess the effect of treatments on the binary variables, appropriate anti-predator reaction and startle due to the small sample size. We tested the effect of trial type using the Mcnemar test on the startle responses.

## Results

**Fig. 3:**
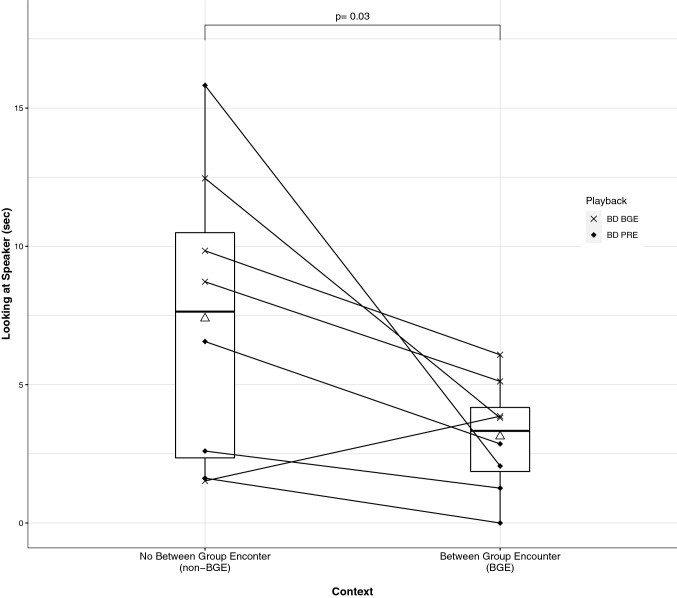
Box plot showing a comparison of duration of looking towards the speaker in two treatments. Solid line in the box denotes the median, Δ denotes the mean, solid dots connected by lines represent raw data points for each subject (*N* = 8) across treatments. Significance of Wilcoxon tests is represented by ‘p’. The call variant used as a playback during the trial is denoted by the shape of the dots

**Fig. 4:**
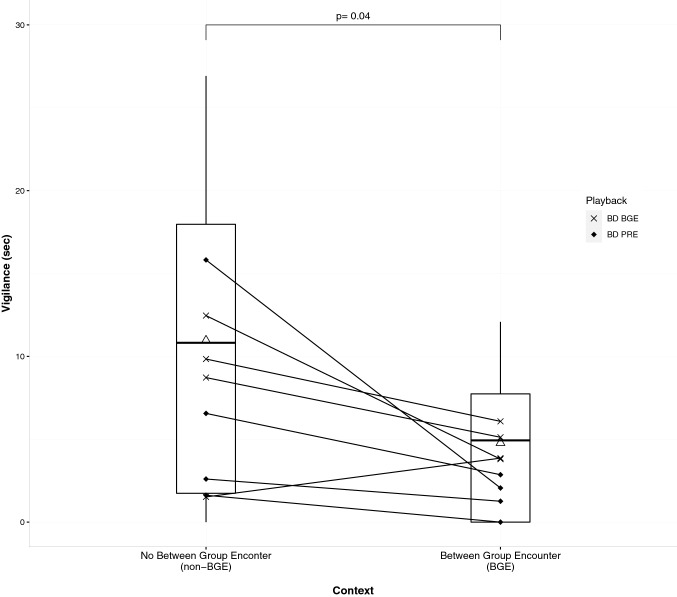
Box plot showing comparison of duration of vigilance behaviour in two treatments. Solid line in the box denotes the median, Δ denotes the mean, solid dots connected by lines represent raw data points for each subject (*N* = 8) across treatments. Significance of Wilcoxon tests is represented by ‘p’. The call variant used as a playback during the trial is denoted by the shape of the dots

Subjects looked toward the speaker for significantly less time in the BGE compared to the non-BGE trials (two-tailed Wilcoxon test (paired): *Z* = 33, *p* = 0.03, *r* = 0.74; Fig. 3). The duration of looking toward the speaker was not affected by the PRE and BGE call variants (two-tailed Wilcoxon test (paired): *Z* = 25, *p* = 0.383, *r* = 0.34). They also showed lower vigilance in BGE compared to non-BGE trials (two-tailed Wilcoxon test (paired): *Z* = 23, *p* = 0.04, *r* = 0.72; Fig. 4). Vigilance was not affected by the PRE and BGE call variants (two-tailed Wilcoxon test (paired): *Z* = 27, *p* = 0.25, *r* = 0.44). Fewer subjects showed startle responses in BGE compared to non-BGE trials (McNemar Test, *p* = 0.04). Startle responses were never observed in the non-BGE trials and in only half of the BGE trials. Additionally, during *N* = 2 BGE trials, the adult male from the other group (AK) responded with alarm barks after hearing the playback of the calls. Predator-specific responses never occurred, neither in the BGE nor in the non-BGE trials.

## Discussion

We investigated the hypothesis that vervet monkeys relied on contextual information to infer the meaning of one of their calls, the male alarm bark, which is given to terrestrial predators, but also during non-predatory intergroup aggression. We were able to show experimentally that recipients reacted differently to the same call, depending on the contexts in which it was presented. When male alarm barks were produced during ongoing intergroup encounters, non-participating subjects were less reactive to the calls, i.e., they looked at the speaker for less time, were less vigilant and never showed startle responses, compared to when the same calls were produced during non-BGE trials.

We selected the variable 'looking towards the speaker' because it was most likely an indication that the subject was looking for information about the reason for the call from the caller (Struhsaker 1967; Seyfarth et al. 1980a). The fact that subjects either looked for less time towards the speaker or one subject did not look when trials were administered during between-group interactions suggested that they had already made interpretations regarding the call. During BGE trials, subjects already had time to learn about the intergroup context, either due to other vocalisations typical for this context or due to having spotted members of the neighbouring group before the playback. Thus, the uncertainty about the cause of the (playback) call was low. In contrast, when the same calls were played during non-BGE trials, subjects had no relevant prior information available about the call's cause.

In BGE trials, low uncertainty also indicates a low likelihood of negative consequences. The subjects were adult females who were away from the frontline of the encounter. Thus, the potential negative consequences were minimal for them. On the other hand, the likelihood of negative consequences could be higher (e.g. ambush by a predator) for the same subjects in the non-BGE trial. Such differences in the likelihood of negative consequences resulting from the uncertainty regarding the cause of the call can increase information-seeking efforts during non-BGE trials.

Another argument for additional information seeking from the caller in non-BGE trials could be a higher rate of false alarms in large groups, which could drive individuals to seek more information before responding, probably to avoid energetic costs (Beauchamp and Ruxton 2007). Furthermore, additional information seeking in a non-BGE context can be used as a strategic response to counter the deceptive alarm calls (Wheeler 2009; Wheeler and Hammerschmidt 2013).

Many subject monkeys spent significantly less time being vigilant or seeking information from the environment during BGE trials. Furthermore, three subjects showed no vigilance during BGE trials (Fig. 4). As the subjects, per design, did not participate in the intergroup encounter and stayed away from the frontline, a reasonable interpretation for them was that the call was directed at a neighbouring group rather than at a predator or themselves. In non-BGE trials, however, no further contextual information was available, which increased the uncertainty about the call's cause and might have resulted in increased vigilance.

Here, it is important to mention a relevant natural observation of aggressive encounter between two other groups in this population made outside this study. On 8th February 2018, the NH and AK groups had an aggressive encounter, and the adult males and other individuals from both groups produced male alarm barks on the frontline. However, during the encounter, a jackal (*Lupulella mesomelas*) was spotted by the observer (AD) stalking a non-participating individual (AD, personal obs.). Several frontline individuals also saw the jackal and produced alarm calls to it, but the non-participating individuals did not react strongly with anti-predator behaviour or vigilance to the alarm calls (as predicted for this context and as documented in the experiment). In this case, the jackal's hunting attempt was unsuccessful as, at the last moment, the targeted monkey saw the jackal and escaped onto a tree. However, the observation highlights a rare situation in which listeners are likely to draw wrong conclusions, which could be fatal. Ambiguous contexts, like the one described here, are probably very rare, such that contextual information is generally reliable.

Finally, startle responses, which are indicative of unexpectedness, were absent during BGE trials but were observed in half of the non-BGE trials. This result is in line with the interpretation that monkeys assumed the presence of a predator in the absence of a disambiguating intergroup context. In other words, the most plausible interpretation is that subjects in non-BGE trials were more affected by an unanticipated male alarm bark than in the intergroup context in which such calls are common. The fact that only some subjects responded this way might be linked to individual differences in age, sex, or social position. As we could not conduct proper statistical analyses for this variable, our results remain suggestive.

Overall, the behavioural responses in the BGE condition indicated that if recipients already had an expectation for why a male alarm bark was produced (in this case, due to the intergroup encounter), they were not specifically affected by the call, in contrast to non-BGE situations where the alarm bark was distinctive with the ongoing context and, in all likelihood, predicted that the male unexpectedly spotted a terrestrial predator. In other words, it appeared that the non-BGE condition in our experiment was perceived as a potential predator encounter. Furthermore, we did not find any support for acoustic variant hypotheses. Although it does not necessarily mean those variants of male alarm barks are acoustically indistinguishable from each other. Perhaps, graded acoustic differences in the call variants (Price et al. 2015) might not be enough to make accurate inferences without considering the external contextual information.

It is essential to note that we never saw predator-specific escape responses, as sometimes seen in response to terrestrial predators. For terrestrial carnivores, the most adaptive anti-predator response is to climb rapidly into the nearest tree or bush (Seyfarth et al. 1980a). Nevertheless, such rapid flight responses have been observed in the study groups, but they typically occur when individuals forage close to each other (AD unpublished data 2019). Thus, these textbook-style antipredator responses may occur primarily under specific circumstances when social contagion overrides individual decision-making (Armstrong 1951; Hoppitt et al. 2007). Similar observations about the general lack of appropriate predator-specific responses and its occurrence due to social contagion were made in a study on a different population of the same species (Ducheminsky et al. 2014). These observations reiterate that responses to alarm calls are not stereotyped as described in earlier studies; i.e. each alarm call type evokes a specific response from the recipients (Seyfarth et al. 1980a). Alternatively, the Amboseli population in Kenya, on which the original study was conducted, was regularly encountered and predated by leopards compared to the population in this study (Robert Seyfarth personal communication). Such differences in predatory pressure among populations could also explain the differences in predator-specific responses to male alarm barks.

Recent similar experiments on closely related green monkeys (*Chlorocebus sabaeus*) are relevant here. During these experiments, researchers primed subjects one hour before the playbacks, which did not have a lasting impact on their subsequent behaviour. In our study, calling context was an ongoing event that may represent a more natural case and explain the diverging results, despite the fact that contextual information could impact long-term responses, albeit weakly (Price and Fischer 2014). Another difference between our study and the green monkey experiment relates to the nature of the playback stimulus (male alarm barks vs female terrestrial predator alarm chirps, respectively). It is possible that male and female calls evoke different responses or serve different functions (Zuberbühler 2005; Mehon and Stephan 2021). The function of the male alarm bark in South African vervet monkeys has not yet been studied systematically. However, the playback call during two BGE trials triggered alarm barks from a male of the opposing group, pointing towards a possibility that these alarm calls also communicate to both predators and conspecific competitors, as noted by previous studies (Cheney and Seyfarth 1992; Price et al. 2015).

Whatever the function of alarm barks might serve, the critical point remains that male alarm barks in all contexts might have predicted the presence of potential threats (although of different types) to receivers. And the contextual information facilitated locating and specifying the source of the threat, allowing individuals to respond appropriately (Deshpande et al. 2022).

Generally, our results can be interpreted as a systematic use of contextual information to assign meaning to primate alarm calls. Similarly, Diana monkey responses to heterospecific alarm calls are based on prior information about probable causes (Zuberbühler 2000b, c). Capuchin monkeys ignore alarm calls given during competitive feeding events. However, they respond strongly to the same calls in other situations (Wheeler and Hammerschmidt 2013), and baboons respond to grunts depending on the context in which they were produced (Rendall et al. 1999). Taken together with these studies, our results suggest primates can take into account social and environmental cues to infer the most probable cause and the potential consequences of call production, i.e., causal inference becomes the basis for the attribution of meaning.

## Supplementary Information

Below is the link to the electronic supplementary material.

Supplementary file1 (DOCX 78 KB)

## Data Availability

Data and the code for the analyses are available on the following link: https://osf.io/kw5qg/?view_only=8ff2cf2f00164c0fbacc6ee4c8e568e5.
