# Supplementary material for: Context-dependent alarm responses in wild vervet monkeys
Source: Anim Cogn. 2023 Mar 17;26(4):1199–208. doi: 10.1007/s10071-023-01767-0 (PMC10345060; doi:10.1007/s10071-023-01767-0)
Supplement: Supplementary file 1 — Supplementary file1 (DOCX 78 KB) [file 10071_2023_1767_MOESM1_ESM.docx]

**Supplementary material :**

**Context-dependent alarm responses in wild vervet monkeys**

Adwait Deshpande^1,2,3,4,*^ Erica van de Waal^2,5^ and Klaus Zuberbühler^1,2,6^

^1^ Department of Comparative Cognition, Institute of Biology, University of Neuchatel, Neuchatel, Switzerland;

^2^ Inkawu Vervet Project, Mawana Game Reserve, KwaZulu-Natal, South Africa

^3^ Department of Collective Behavior, Max Planck Institute of Animal Behavior, Konstanz,

Germany

^4^ Centre for the Advanced Study of Collective Behaviour, University of Konstanz,

Konstanz, Germany.

^5^ Department of Ecology and Evolution, University of Lausanne, Lausanne, Switzerland

^6^ School of Psychology and Neuroscience, University of St Andrews, St Andrews, United Kingdom

**Call recording and acoustic analyses**

We recorded alarm barks from 13 known adult males and two unknown males from the three neighbouring groups (NH, BD, AK). The calls were recorded in two contexts in which they were produced (1) During aggressive between-group encounters (BGE calls) and (2) During encounters with terrestrial predators (PRE calls)., which include jackals (*Lupulella mesomelas*) and feral dogs. To record the calls, we used a solid-state audio recorder (Marantz PMD 661) and a directional microphone (Sennheiser MKH 416 P48) with a sampling frequency of 44.1 kHz

Call bouts contained call elements separated by at least 500 ms. We extracted a total of 62 bark elements recorded from two contexts (BGE: N= 28, PRE: N= 34). To extract acoustic parameters, the bark elements were manually marked using spectrograms in Raven pro 1.5 (Center for Conservation Bioacoustics, 2014) using Hamming window at 1024 DFT and 93.8% overlap.

We initially extracted 17 acoustic parameters (Table S1) and reduced dimensionality by removing highly correlated (>0.95) acoustic parameters. The remaining 12 were then used to estimate the acoustic similarity between calls recorded in different contexts.

First, we scaled all the remaining parameters and visually checked if they fit normal distribution using quantile-quantile plots (Figure S1). We conducted two-sided T-tests with Bonferroni correction for each of the five normally distributed acoustic parameters to evaluate the difference between the BGE and PRE calls. We found no statistically significant difference for any of the acoustic parameters. (Figure S2 and Table S2). For the remaining seven acoustic parameters, we conducted non-parametric two-tailed Wilcoxon tests with Bonferroni correction. We found no statistically significant difference for any of the acoustic parameters in this category except for the average entropy* (Figure S3 and Table S3)

Our analyses suggest that the male alarm bark variants produced in two contexts, BGE and PRE, cannot be easily differentiated acoustically. We acknowledge that the acoustic analyses conducted in this study are straightforward. However, our results are further supported by an earlier study on the male alarm barks of the same population. Besson et al. recorded 248 male alarm barks from the BGE and PRE contexts from the same groups in 2017 (Besson, 2017). The authors conducted permuted discriminant function analysis (pDFA) to test the difference between male alarm barks produced during BGE and PRE contexts. They found no significant acoustic difference between the two call variants produced in BGE and PRE contexts. Furthermore, a separate independent study has reached a similar conclusion. Price et al. also suggested that male alarm barks between two contexts show high acoustic overlap (Price et al., 2015). Overall, the three independent analyses suggest that terrestrial predator calls produced during BGE and PRE are acoustically highly similar and could be classified as single call types with a graded variation.

*The average entropy in a selection is calculated by finding the entropy for each frame in the selection and then taking the average of these values. Unlike the aggregate entropy which uses the total energy in a frequency bin over the full time span, the average entropy calculates an entropy value for each slice in time and then averages. As a result, the average entropy measurement describes the amount of disorder for a typical spectrum within the selection (Definition from RAVEN software user manual)

**Supplementary GLMM analyses:**

To cross-verify the results of our non-parametric statistics and to investigate the effects of other variables such as audience size and trial order, we constructed linear mixed effect models (LME) using the lme4 (Bates et al., 2015) package in R. For LMEs, we log-transformed the response variables 'looking towards the speaker' and 'vigilance' to fit the assumptions of normal distribution. We created full models for both response variables by adding trial type (BGE vs non-BGE trials), call variant (BGE vs PRE), audience size, and trial order as fixed effects. We added subject identity as a random effect in the full models to control for repeated testing.

For model selection, we used an information-theoretic approach. We created two sets of candidate models corresponding to the two full models. Candidate models for each set were created by removing one fixed effect at a time from the full models, finally achieving null models without any fixed effect. We then compared the Akaike information criterion (AIC) values of all the models in the given set. We retained a final model from each set where AIC values increased by at least 2.0. In a post-hoc analysis, we checked Akaike's information criterion. We corrected for small sample sizes (AICc) values and AICc weights of the two selected final models compared to all other candidate models within the sets using the 'model. sel' function in the MuMIn package (Barton, 2009). The comparison revealed that both the final models had minimum AICc values and the highest AICc weights in the corresponding set. Zero inflation combined with a small sample size prevented us from using LMEs for the startle responses.

**GLMM Results:**

Subjects looked toward speaker for significantly less time in the BGE compared to the non-BGE trials (LME Estimates (log-transformed) = - 0.63 ±0.24, p= 0.008, ΔAIC= - 2.48; Table S4). They also showed lower vigilance in BGE compared to non-BGE trials (LME: Estimates (log-transformed) = 0.50 ±0.18, p= 0.006, ΔAIC= - 2.15; Table S5)

**References**

Barton, K. (2009). *MuMIn: Multi-model inference* (1.43.17). https://cran.r-project.org/package=MuMIn

Bates, D., Maechler, M., Bolker, B., & Walker, S. (2015). Package lme4. *Journal Of Statistical Software*, *67*(1), 1–91. https://doi.org/http://lme4.r-forge.r-project.org

Besson, E. (2017). *Vervet monkeys alarm calls: context specific or not?* Dissertation [University of Neuchatel].https://www.unine.ch/files/live/sites/compcog/files/Thesis/170828_Msc_Thesis_BESSON_Emmanuelle.pdf

Center for Conservation Bioacoustics. (2014). *Raven Pro: Interactive Sound Analysis Software* (1.5). The Cornell Lab of Ornithology. http://ravensoundsoftware.com/

Price, T., Wadewitz, P., Cheney, D. L., Seyfarth, R. M., Hammerschmidt, K., & Fischer, J. (2015). Vervets revisited: A quantitative analysis of alarm call structure and context specificity. *Scientific Reports*, *5*(13220), 1–11. https://doi.org/10.1038/srep13220


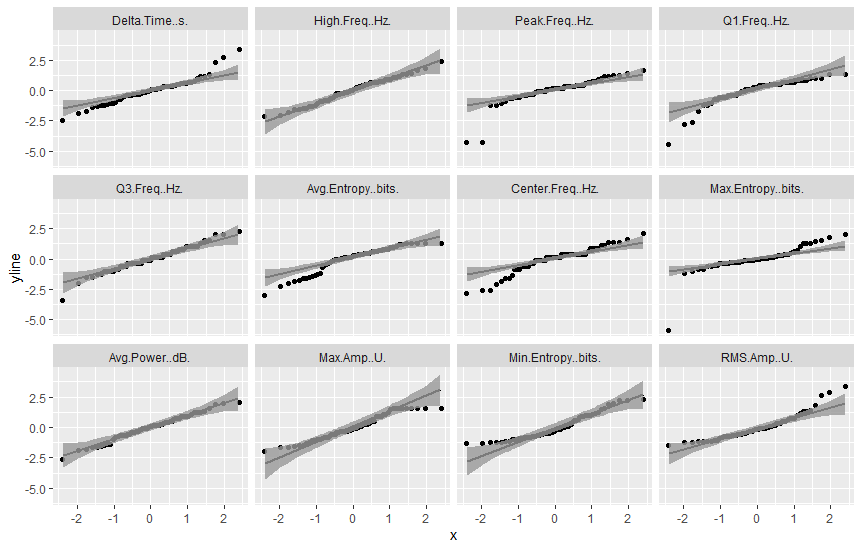


Figure S1: Quantile-quantile plots of the acoustic parameters used for the analyses. The line shows theoretical values for normal distribution with a 95% confidence interval band around it. Through visual inspection, we selected the following parameters as normally distributed 1. Avg Power; 2. High Freq; 3.Max Amp; 4.Min Entropy; 5. Q3 Freq


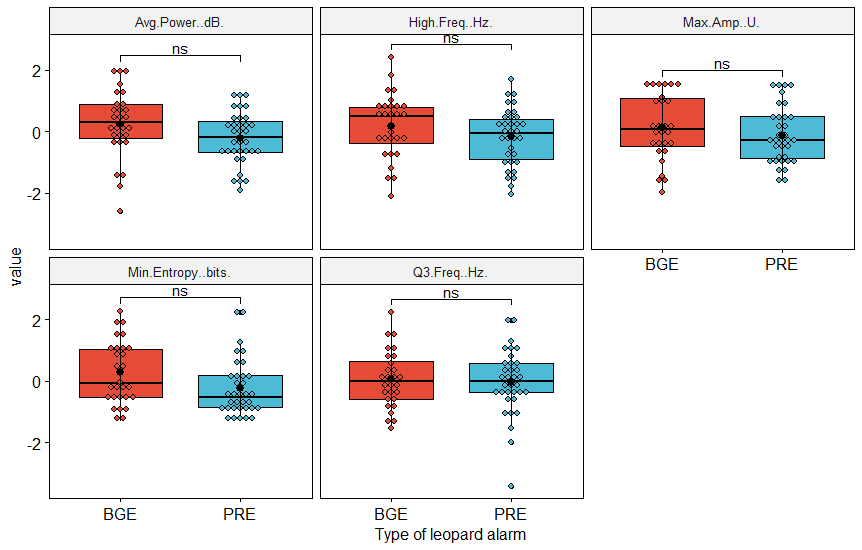


Figure S2: Each panel show boxplot of an acoustic parameter (scaled data) for two variants of male alarm barks (BGE and PRE). The whiskers denote the 1st and third quartiles. The mean is marked with a black dot, and the median with a solid black line. Coloured dots show data points. 'ns' denotes non-significance for the T-tests


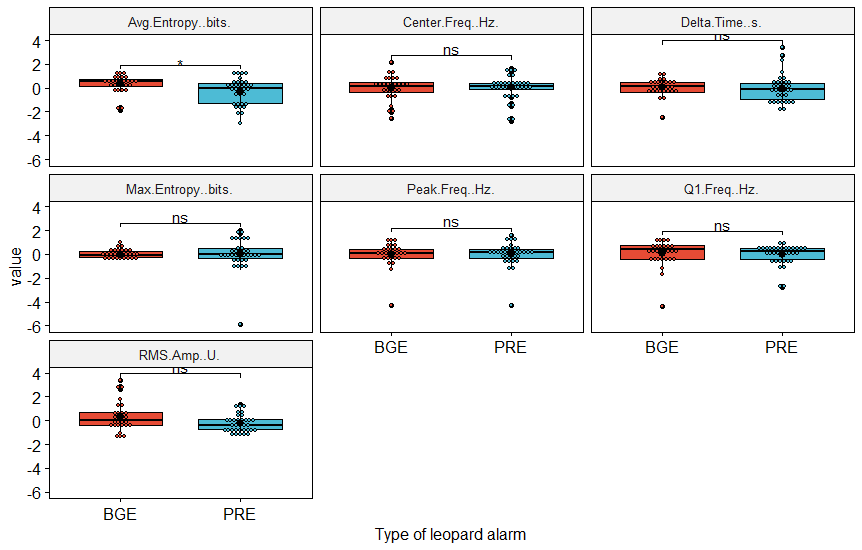


Figure S3: Each panel show boxplot of an acoustic parameter (scaled data) for two variants of male alarm barks (BGE and PRE). The whiskers denote the 1st and third quartiles. The mean is marked with a black dot, and the median with a solid black line. Coloured dots show data points. 'ns' and'*' denotes non-significance and significance for the Wilcoxon tests

Table S1: List of acoustic parameters extracted from the calls. The highlighted parameters were used for further analyses. Highly correlated parameters are shown in white which were excluded from the analyses.

| **Acoustic Parameter (Unit)** | **Acronym** |
| --- | --- |
| High Frequency (Hz) | High.Freq..Hz. |
| Duration (s) | Delta.Time..s. |
| Peak Frequency (Hz) | Peak.Freq..Hz. |
| 1st Quartile Frequency (Hz) | Q1.Freq..Hz |
| 3rd Quartile Frequency (Hz) | Q3.Freq..Hz. |
| Delta Frequency (Hz) | Delta.Freq..Hz |
| Average Entropy (bits) | "Avg.Entropy..bits |
| Center Frequency (Hz) | Center.Freq..Hz |
| Max Entropy (bits) | Max.Entropy..bits. |
| Average Power (dB) | Avg.Power..dB. |
| Max Amplitude (U) | Max.Amp..U |
| Min Entropy (bits) | Min. Entropy..bits. |
| Energy (dB) | Energy..dB |
| Peak Amplitude (U) | Peak.Amp..U. |
| Peak Power (dB) | Peak.Power..dB |
| Root-mean-square Amplitude | RMS.Amp..U |
| Sound Exposure Level (dB) | SEL..dB |

Table S2: T-test statistics for five acoustic parameters of two call variants. (ns= non-significant)

| *Acoustic parameters* |  | *Call variant 1* | *Call variant 2* | *T test statistic* | *Degrees of freedom* | *P value* | *Adjusted p-value* | *Significance* |
| --- | --- | --- | --- | --- | --- | --- | --- | --- |
| Avg.Power..dB. |  | BGE (n=28) | PRE (n=34) | 1.8 | 49.77 | 0.08 | 0.39 | ns |
| High.Freq..Hz. |  | BGE (n=28) | PRE (n=34) | 1.36 | 54.17 | 0.18 | 0.9 | ns |
| Max.Amp..U. |  | BGE (n=28) | PRE (n=34) | 0.94 | 53.8 | 0.35 | 1 | ns |
| Min. Entropy..bits. |  | BGE (n=28) | PRE (n=34) | 2.01 | 55.25 | 0.05 | 0.25 | ns |
| Q3.Freq..Hz. |  | BGE (n=28) | PRE (n=34) | 0.33 | 59.85 | 0.74 | 1 | ns |

Table S3: Wilcoxon test statistics for seven acoustic parameters of two call variants (ns= non-significant, * = significant)

| *Acoustic parameters* | *Call variant 1* | *Call variant 2* | *Wilcoxon test statistic* | *p* | *p adjusted* | *p adjusted significance* |
| --- | --- | --- | --- | --- | --- | --- |
| Avg.Entropy..bits. | BGE (n=28) | PRE (n=34) | 681 | 0 | 0.03 | * |
| Center.Freq..Hz. | BGE (n=28) | PRE (n=34) | 466.5 | 0.9 | 1 | ns |
| Delta.Time..s. | BGE (n=28) | PRE (n=34) | 545 | 0.33 | 1 | ns |
| Max.Entropy..bits. | BGE (n=28) | PRE (n=34) | 468.5 | 0.92 | 1 | ns |
| Peak.Freq..Hz. | BGE (n=28) | PRE (n=34) | 452 | 0.74 | 1 | ns |
| Q1.Freq..Hz. | BGE (n=28) | PRE (n=34) | 523 | 0.51 | 1 | ns |
| RMS.Amp..U. | BGE (n=28) | PRE (n=34) | 619 | 0.04 | 0.3 | ns |

Table S 4: Set of candidate models for the response variable (looking towards the speaker; log-transformed; LLS). Treatment= Non-BGE vs BGE; Call variant= BGE vs PRE; Audience.5m= number of group members within 5m of a subject during the playback; Order= Order of the trial. The subject's identity was added as a random effect in each model. The final model is highlighted in bold, where AIC values increased by more than 2.0

| **Model** | **DF** | **AIC** | **Δ AIC** |
| --- | --- | --- | --- |
| LLS ~ Treatment + Call variant + Audience.5m. + Order + (1\|Subject) | 7 | 46.5 | 0.00 |
| LLS ~ Treatment + Call variant + Order +(1\|Subject) | 6 | 43.14 | -3.36 |
| LLS ~ Treatment + Call variant + (1\|Subject) | 5 | 39.25 | -3.89 |
| **LLS ~ Treatment + (1\|Subject)** | **4** | **39.37** | **0.12** |
| LLS ~ 1 + (1\|Subject) | 3 | 41.85 | +2.48 |

Table S5: Set of candidate models for the response variable of vigilance (log-transformed; LVIG). Treatment= Non-BGE vs BGE; Call variant= BGE vs PRE; Audience.5m= number of group members within 5m of a subject during the playback; Order= Order of the trial. The subject's identity is added as a random effect in each model. The final model is highlighted in bold, where AIC values increased by more than 2.0

| **Model** | **DF** | **AIC** | **Δ AIC** |
| --- | --- | --- | --- |
| LVIG ~ Treatment + Call variant + Audience.5m. + Order + (1\|Subject) | 7 | 49.29 | 0.00 |
| LVIG ~ Treatment + Call variant + Order +(1\|Subject) | 6 | 44.46 | -4.84 |
| LVIG ~ Treatment + Call variant + (1\|Subject) | 5 | 39.8 | -4.66 |
| **LVIG ~ Treatment + (1\|Subject)** | **4** | **39.62** | **-0.18** |
| LVIG ~ 1 + (1\|Subject) | 3 | 41.77 | +2.15 |
